# Pathogen Sensors

**DOI:** 10.3390/s91108610

**Published:** 2009-10-28

**Authors:** Joseph Irudayaraj

**Affiliations:** Department of Agricultural and Biological Engineering, 225 S. University Street, Purdue University, West Lafayette, IN 47907, USA; E-Mail: josephi@purdue.edu; Tel.: +1-765-494-0388; Fax: +1-765-496-1115

The development of sensors for detecting foodborne pathogens has been motivated by the need to produce safe foods and to provide better healthcare. However, in the more recent times, these needs have been expanded to encompass issues relating to biosecurity, detection of plant and soil pathogens, microbial communities, and the environment. The range of technologies that currently flood the sensor market encompass PCR and microarray-based methods, an assortment of optical sensors (including bioluminescence and fluorescence), in addition to biosensor-based approaches that include piezoelectric, potentiometric, amperometric, and conductometric sensors to name a few. More recently, nanosensors have come into limelight, as a more sensitive and portable alternative, with some commercial success. However, key issues affecting the sensor community is the lack of standardization of the testing protocols and portability, among other desirable elements, which include timeliness, cost-effectiveness, user-friendliness, sensitivity and specificity.

This special issue, while focused primarily on “Pathogen Sensors”, covers a range of sensing processes and technologies relevant to microbes in general. While the demand for portable, onsite sensors for rapid detection will continue to be on the rise because of the needs in the food, agriculture, healthcare, environmental, and bio-defense sectors, the onus is on the industries and research institutions to continue to partner and innovate better products.

Fundamentally, immunoreaction assessed/sensed by one of the physical mechanisms could constitute biosensing, in a general sense. However, given the space and scope, I have attempted to compartmentalize the reports submitted to this special issue based on the following broad categories:

*Nanoscale and microscale approaches* with emphasis on microfluidic platforms for pathogen and pathogenesis assessment. A critical review on the limitations of antibody-based biosensors is provided [1]. This is followed by reviews on current strategies in pathogen sensing with an emphasis on nanoscale, lab-on-chip, microfluidics, and array technologies to provide a broader understanding and exposure to this field [2,3].

*Microbial biosensors* that capitalize on the molecular processes and mechanisms for sensor development. Detection of amino acid bioavailability, fabrication of membrane engineered structures for virus detection [4], multivalent anchoring of antibodies on cellulose as platforms for pathogen sensors are discussed [5] in addition to a report on Gram-negative bacteria sensors for eukaryotic signaling [6].

A segment on *Biosensors and traditional methods* with an introduction to electroanalytical sensors for multiplex pathogen detection is included [7]. Topics discussed in this category include immunomagnetic bead capture and antibody microarrays [8,9] and PCR and DNA-based assays [10] for detection in food and feed [11] respectively.

*Other complementary approaches* include articles that relate to the use of microbial films [12,13], acoustic wave biosensors [14], electronic nose [15] and waveguide -based biosensors [16].

In conclusion, I anticipate pathogen sensor development efforts to continue to play a crucial role in ensuring safe foods as well as in early detection, as a prognostic measure and for routine health monitoring. Amidst hurdles, the race to produce the most effective biosensor will persist, because of the excitement and challenge to fulfill a critical societal need.
